# GP emigration from Ireland: an analysis of data from key destination countries

**DOI:** 10.1186/s12913-024-12117-2

**Published:** 2024-12-20

**Authors:** Holly Rose Hanlon, Éidín Ní Shé, John-Paul Byrne, Susan M. Smith, Andrew W. Murphy, Aileen Barrett, Mike O’Callaghan, Niamh Humphries

**Affiliations:** 1 Graduate School of Healthcare Management, RCSI University of Medicine and Health Sciences, 111 St. Stephen’s Green, Dublin, Ireland; 2 RCSI University of Medicine and Health Sciences, Dublin, Ireland; 3 Trinity College Dublin, Dublin, Ireland; 4 University of Galway, Galway, Ireland; 5 Irish College of General Practitioners, Dublin, Ireland

**Keywords:** Primary care, Medical workforce, Medical migration, Health system research, Ireland

## Abstract

**Background:**

Ireland is experiencing a general practitioner (GP) workforce crisis, facing an ageing workforce, a growing population with increased life expectancy, and increased complexity of patients. The GP crisis threatens access to primary care in Ireland, as well as Ireland’s aim to transform into a primary-care centred system of universal healthcare via the proposed “Sláintecare” healthcare reforms. The challenges faced are common to many countries as health systems seek to expand their medical workforce post-pandemic. In addition Ireland has a legacy of austerity policies which impacted the health system, and triggered/generated largescale doctor emigration. However, little is known specifically about *GP emigration* and the role it potentially plays in the GP workforce crisis. This paper aims to address the gap in knowledge about the level of GP emigration from Ireland and consider the implications for the Irish health system and health systems internationally.

**Methods:**

As Ireland does not formally collect routine data on GP emigration, this paper presents routinely collected secondary data from four key destination countries; Australia, New Zealand, the United Kingdom, and Canada, in order to gain an initial picture of GP emigration from Ireland to these countries, from 2012–2021. The data were in the form of medical registration and immigration (visa) data and both stock (the total number of GPs registered in a country in a given year) and flow data (the number of GPs entering a country in a given year) were collated, where available.

**Results:**

The stock data shows a substantial cohort of Irish-trained doctors working in general practice in key destination countries. The flow data suggests a relatively small annual emigration flow of GPs from Ireland to individual countries. However when compared with the total numbers of GPs trained in Ireland each year, the numbers are notable.

**Conclusions:**

The available data suggests a mixed picture regarding GP emigration from Ireland. There is a significant stock of Irish-trained GPs abroad which perhaps represents a potential cohort of GPs who could be encouraged to return to practice in Ireland as part of Ireland’s strategy for addressing the GP workforce crisis. The annual flow of GPs from Ireland to key destination countries, while small, should be monitored and factored into GP workforce planning. As global demand for GPs increases, countries will inevitably compete with each other to attract and retain GPs (see for example Australia’s recent move to attract and recruit Irish trained GPs). The paper highlights the need for improved routine data on the GP workforce in Ireland, including the need for a national GP workforce dataset, in order to ensure that national workforce planning efforts are informed by the latest evidence on GP emigration.

## Background

Primary care is the foundation of a strong healthcare system that ensures positive health outcomes and health equity [1]. The World Health Organisation (WHO) has long advocated for primary care as a central component of a successful health system [2] as primary care is rooted in communities and seeks to address the health problems of individuals by integrating care, prevention, promotion and education [3, 4].

Primary care systems can vary in terms of the services provided, how those services are financed and delivered and the access cost (to patients). The mix of disciplines within a primary care workforce also vary from country to country but general practice/family medicine is considered the cornerstone of primary care [5]. General practice/family medicine is a “clinical specialty oriented to primary care” [4], and the terms “general practitioner” and “family doctor” are used interchangeably depending on country of context; with the term “primary care physician” also used in the United States [4, 6]. As general practitioner (GP) is the commonly used term in Ireland, this is the term used throughout this paper to denote primary care doctors. The Irish College of General Practitioners (ICGP) has defined a GP as “a medical graduate who gives personal, primary and continuing care to individuals, families and a practice population, irrespective of age, gender and illness” [7].

Ireland faces a GP workforce crisis. In 2022 the Irish College of GPs stated that Ireland “*cannot meet the current or future GP workforce or workload demands*” noting that it is becoming more difficult “*to find replacements for retiring GPs, or new GPs to expand their practices and deal with growing workloads*” [8]. Ireland is not alone in facing these challenges, the GP workforce is also in crisis globally, with issues in the US, Australia, New Zealand, and across the European region [6, 9], with staff shortages posing a threat to doctor wellbeing and patient safety [10]. The drivers for the GP workforce crisis are varied and complex. The World Health Organisation’s Time to Act report highlighted an ageing workforce, insufficient recruitment, problems with retention, and increased mobility of healthcare workers, including emigration, as major issues for the health and care workforce across Europe [11]. Perhaps the most straightforward contributing factor is the increased proportion of the GPs workforce approaching retirement age [12, 13] and the failure of workforce planning to train an adequate numbers of replacement GPs [14]. A 2021 report on medical staffing levels in England found that retention of doctors overall is poor, and that not enough doctors are being trained to meet increased healthcare demand [14]. In general practice, researchers found that there were 4% fewer qualified full-time equivalent GPs in the UK in 2021 than in 2015 and that there had been a 22% increase in GP patient visits during that time [14]. Canada reports similar staff shortages and notes that one in six Canadians lack a regular GP [15]. In the UK, general practice is described as being “on its knees” [16]. One UK survey found that almost 20% of GPs intended to leave general practice or retire in the next two years [17]. There have also been significant increases in the number of UK GPs registering to work abroad [18] and NHS GPs in 2024 announced industrial action in England [19, 20].

Another driver of the GP workforce crisis is the decreased attractiveness of general practice as a career [21] and the working conditions created by the GP crisis itself. This is starkly summarised in a 2022 Commonwealth Fund survey of GPs in 10 high-income countries, which found that the majority of those surveyed were not satisfied with their medical practice, their income, the amount of time spent with patients, their workload, the amount of time spent on administrative work, or with their work-life balance [9].

The COVID-19 pandemic has also had an impact on the GP workforce. General Practitioners (GPs) were central to the pandemic response playing a vital role in the vaccination rollout [22] and in the pandemic response more generally. While the acute stage of the COVID-19 pandemic has passed, GPs continue to work under pressure as they deal with the backlog of work produced by delayed care during the pandemic [23]. Going forward, the legacy of the pandemic and its ongoing consequences for the population (mental health impact, effects of long-COVID) will continue to be felt by GPs [22].

### The Irish context

Even before the pandemic, GPs felt that “*financial cuts during the economic crisis…brought general practice in Ireland close to collapse*” [24]. The challenges faced by Ireland in relation to primary care are faced by many developed countries; population increases, increased life expectancy and increased complexity of patients, without a corresponding increase in GP numbers [25]. A further risk in the Irish context is in 2022 it was reported that 24% of Ireland’s GPs were due to retire in the coming decade [25]. The 2018 Health Service Capacity review projected a need for an additional 1400 WTE GPs by 2031, a figure that did not account for the “large number” of GPs expected to retire by that date [26]. The GP workforce crisis is particularly severe in rural Ireland, where retiring GPs are struggling to recruit replacements, and practicing GPs are unable to find locum cover to enable them to take annual leave or sick leave [25].

Ireland’s GP workforce crisis has also been shaped by the austerity policies and health budget cuts which followed the 2008/2009 economic crash [27]. These budget cuts reduced GP income [28, 29]. They also impacted GPs in their role as gatekeepers [30] to the health systemas patient waiting times for specialist treatment lengthened significantly [31]. almost 700,000 patients on hospital waiting lists [32] awaiting specialist care. These patients require ongoing GP care while they wait for their hospital appointments and this contributes to the intensification of GP workloads [33].

The GP crisis has implications for the wider health system; specifically in relation to Ireland’s ability to successfully implement the “Sláintecare” healthcare reforms, which aim to increase access to public healthcare in Ireland, and to expand primary and community healthcare [30].

In 2020 it was predicted that Ireland would need to expand its GP workforce by 32–42% by 2028 [27]; to achieve this, the government increased the number of GP training places from 186 in 2020 to 285 in 2023 [34] and extended the age of GP retirement [35]. It has also increased funding for general practice, via the Chronic Disease Management programme, the rollout of free contraceptive services, and the extension of free GP services to children aged 7 and under, among other investments [36].

While more attention has been paid to increased training of GPs in recent years, less attention has been given to the issue of GP retention, with minimal data or research to date on GPs’ career paths post- graduation. There has been recent progress on this front; the HSE NDTP medical workforce planning team published an Annual Medical Retention Report for 2023 [37] which included a section on GP retention. It noted that of the GPs who qualified in Ireland from 2016–2021, 87% reported working as a GP in Ireland in 2022 [37]. This indicates that 13% of GP graduates 2016–2021 were not working in general practice in Ireland by 2022. Further research and data collection into this cohort would enable the Irish health system to better understand the career and emigration pathways of Irish-trained GPs post-graduation and could inform GP workforce planning.

### Doctor emigration from Ireland

Emigration has been a major contributor to the challenges facing the Irish health workforce generally; secondary data analysis has shown that Ireland has a high rate of doctor emigration [38]. Indeed, OECD research refers to “The Irish Paradox” that despite Ireland training a relatively high number of Irish and foreign doctors (half of Ireland’s medical students are from outside of the EU [39]), Ireland nonetheless relies heavily on international recruitment to staff the health system [40]. This reliance is due in part to high levels of outward migration of doctors [40]. As of 2016, Ireland had the highest proportion of internationally trained doctors in the EU, at 42% [40]. This has reduced slightly to 40% in 2021 [41] and to 39.4% in 2023 [42]. Similar situations can be found in other countries; for example, in Romania, which trains a large number of doctors, but has one of the lowest rates of doctors per population in the EU, in part due to migration [43]. Shortages of doctors are a global issue, and health worker migration has ethical implications particularly in relation to the migration of doctors from developing to developed countries and the implications for equitable access to healthcare [44]. The high proportion of Irish and Irish-trained doctors emigrating from Ireland has clear implications for other countries as it continues to fuel Ireland’s need for internationally trained doctors(e.g. 71% of new entrants to the Irish medical register in 2022 were internationally-trained [45]). In terms of inward migration of doctors into GP practice, over 100 international doctors have already been recruited to work in rural GP practices in Ireland under the International Medical Graduate Rural GP Programme [46].

However, little is known about GP emigration *from* Ireland, and the extent to which it may impact on the GP workforce crisis. Previous HSE predictions from 2015 predicted that a GP emigration rate of 15% would necessitate training an additional 75–138 more GPs each year [47]; but the actual annual rate of GP emigration remains unknown. This paper will attempt to quantify GP emigration from Ireland by presenting secondary data on GP emigration from Ireland to Australia, the United Kingdom, Canada, and New Zealand (which are the key destination countries for doctors emigrating from Ireland [48]). As global demand for GPs increases, high income countries will compete with each other to attract and retain GPs (see for example Australia’s recent move to facilitate the recruitment of Irish-trained GPs [49]). Source countries, such as Ireland, must get better at monitoring and responding to emerging trends in GP emigration and factoring them into GP workforce planning models and policies.

### The GP workforce in Ireland

In order to understand GP emigration from Ireland in a meaningful context, we must first know how many GPs complete training in Ireland every year, as well as the total number of GPs working in general practice in Ireland (i.e., the “stock” of GPs). GP training in Ireland generally takes an additional four years of specialist training following completion of a primary medical qualification [50] but may take longer as doctors may not enter GP training directly [51]. To provide context for the data on GP emigration, we will first present data collated from existing literature on the GP workforce in Ireland.

The number of GP training places has increased substantially in recent years; from 186 in 2020, to 182 in 2021, to 221 in 2022 [52], 285 in 2023 [34] and 350 in 2024 [34].

HSE NDTP data shows the number of GPs graduating in Ireland each year for the past five years; see Table 1 [52]. These numbers will increase in coming years in line with the increased number of GP training places (outlined above).

**Table 1:** Number of qualified GPs graduating in Ireland each year ([52])

**2018**	**2019**	**2020**	**2021**	**2022**
152	141	155	144	182

Obtaining a picture of the overall stock of GPs in Ireland is more complex, as there is no central register of GPs in practice in Ireland. There are several sources with varying but relatively similar numbers (see Table 2).

**Table 2:** Stock of GPs in Ireland according to various sources (ICGP [53], Oireachtas (government) website [54], Irish Medical Council (IMC) [45])

Source	Number of GPs
ICGP (2023)	4200
Oireachtas (government) website (2022)	4319
Irish Medical Council (MCI) (2022)	4953

The three sources measure something slightly different. The figure from the Irish Council of GPs relates to the number of practicing GPs who are also members of ICGP (approximately 85% of practicing GPs in Ireland [53]). It should be noted that membership of ICGP is optional for GPs. The Oireachtas (government) website cites the number of GPs on the specialist division of the medical register whose registered speciality is general practice. However, this does not include those GPs who work in general practice but without a specialist qualification in general practice. The data also fails to indicate whether or not all of these GPs are currently practising as GPs, or whether they are full or part time GPs [54] Data from the Medical Council of Ireland (MCI) 2022 [45], shows that among the clinically active doctors on the register, 4953 doctors reported general practice as their current area of practice. Of the doctors working in general practice, 4431 self-reported their specialty as general practice, and of these, 3572 were on the specialist division of the medical register, while 797 were on the general division of the register and 62 were trainee specialists [45]. This complexity with the stock data, highlights the need for a centralised database of clinically active doctors working in general practice, so that medical workforce planners have access to an accurate dataset on the current GP workforce. The need for a register of GPs was previously highlighted by HSE NDTP in 2015 [47].

For the purposes of this paper we will refer to the MCI figure of 4,953 as the number of doctors on the medical register and who self-report that they are working in general practice in Ireland [45].

## Methods

### Study design

This is an observational study using routinely collected data drawn from seven sources across four key destination countries for which data were readily available, in order to develop an insight into GP emigration from Ireland. No ethical review process was required for this paper as it used routinely collected, anonymised aggregate data, which were publicly available.^1^

As Ireland does not collect any formal routine data on GP emigration, secondary data on GP migration were collated from Australia, New Zealand, the United Kingdom, and Canada. Data were obtained from professional medical registers and/or immigration records in the destination countries. We collated both stock and flow data wherever possible (stock data: the total number of GPs registered with medical councils in a country in a given year; flow data: the number of GPs entering a country in a given year via immigration visas issued and/or the numbers of new entrants to medical registers). This is an established method of collecting health workforce data [55, 56]. Because of the nature of the data, the stock data relates to Irish-trained doctors, i.e. doctors with a primary medical qualification (PMQ) from Ireland and the flow data relates to either Irish-trained doctors, or Irish citizen doctors, depending on whether medical registration or immigration data were used.

**Fig. 1:**
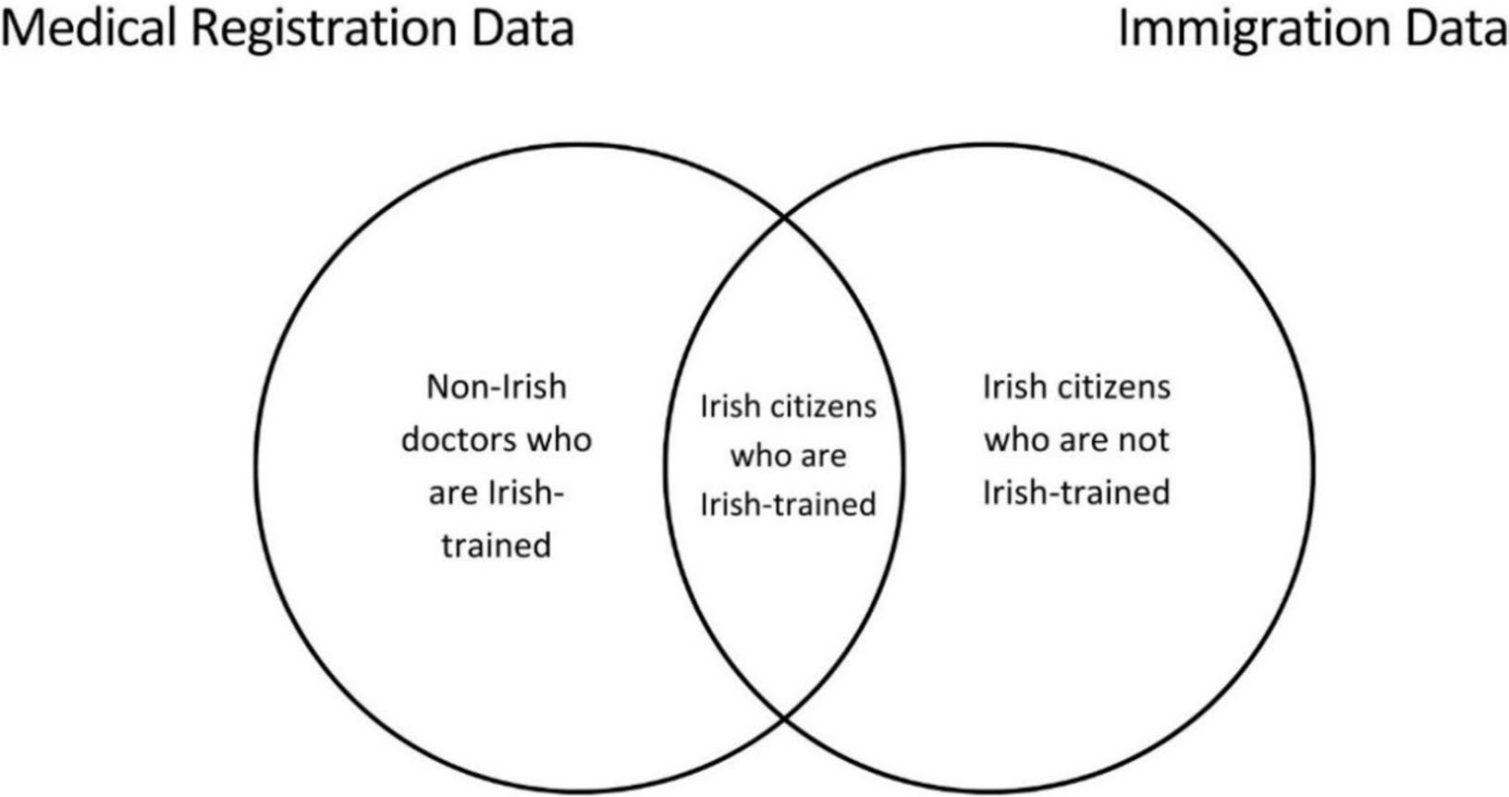
Graphic representation of cohorts of Irish doctors abroad as captured by different types of data

The complexity of the specific groups captured by each type of data source (medical registration versus immigration data) is outlined in Fig. 1. Essentially, Irish doctors abroad are comprised of three separate cohorts.

- Irish citizen doctors who are also Irish-trained (PMQ from Ireland)
- Irish citizen doctors who are not Irish-trained (PMQ from outside Ireland)
- Non-Irish doctors who are Irish-trained (PMQ from Ireland)

The two different formats of data (stock and flow) differ in which of these cohorts they capture, depending on the type of data (medical registration versus visa data) provided (see Fig. 1 above).

Where available, data were also collected on the number of Irish-trained doctors enrolled in GP training programmes in the destination countries. This data was used in an attempt to understand discrepancies between the stock and flow data collected for New Zealand (discussed in further detail in the Results section). We also provide an overview of the current GP workforce in Ireland to put this emigration data into context.

Specific sources for the data obtained from each country are outlined in Table 3 below. With the exception of the Australian visa data, which was obtained from the Australian Department of Home Affairs Temporary Work (skilled) visa program website [57], all data was requested and obtained directly from the sources via email.

**Table 3:** Sources of data on GP emigration from Ireland to key destination countries

Country	Stock Data	Flow Data	Data on Irish-Trained Doctors Enrolled in Training Abroad
Australia	National Health Workforce Dataset on Medical Practitioners, Department of Health and Aged Care	Department of Home Affairs [57]	Royal Australian College of General Practitioners
New Zealand	New Zealand Medical Council	New Zealand Medical Council	New Zealand Medical Council
United Kingdom	General Medical Council	General Medical Council	General Medical Council
Canada		Immigration, Refugees & Citizenship Canada	-

## Results

### Stock data

#### Australia

Table 4 presents the total stock of Irish-trained GPs who were registered to work as GPs in Australia (2014–2021). The stock of Irish-trained GPs in Australia is high and has remained relatively stable (at above 300) since 2014 (the first year for which data was available), with a peak in 2018 before a slight decline 2018–21. Perhaps this indicates a historical pattern of Irish trained GPs emigrating to Australia.

**Table 4:** Number of Irish-trained doctors registered as GPs in key destination countries (stock data) 2012–2021

Country	2012	2013	2014	2015	2016	2017	2018	2019	2020	2021
Australia^a^	-	-	344	353	364	369	379	336	331	313
New Zealand^b^	26	28	33	34	45	41	45	52	53	60
United Kingdom^c^	990	953	889	840	813	787	756	718	710	704
**Total**	**1016**	**981**	**1266**	**1227**	**1222**	**1197**	**1180**	**1106**	**1094**	**1077**

^a^National Health Workforce Dataset on Medical Practitioners, Department of Health and Aged Care

^b^New Zealand Medical Council

^c^General Medical Council

#### New Zealand

Table 4 also outlines the total number of Irish-trained GPs registered with the New Zealand Medical Council. Although low, the stock of Irish-trained GPs in New Zealand has steadily increased since 2012, from 26 in 2012 to 69 in 2021.

#### United Kingdom

Table 4 also shows the total number of Irish-trained GPs registered with the General Medical Council in the UK. The stock of Irish-trained GPs in the UK is very significant, but has decreased from 990 in 2012 to 704 in 2021. The authors speculate that this may relate to historical patterns of migration between the UK and Ireland and the recent reduction in numbers may indicate an Irish trained GP workforce now reaching retirement age.

### Flow data

#### Australia

The number of temporary working visas issued to Irish citizen GPs entering Australia each year from 2012–2022 is outlined in Table 5. From 2012 to 2018 approximately 60–80 Irish GPs emigrated to Australia. This pattern declined sharply in 2019, a trend which continued to date. The authors speculate that this decline could potentially be the result of increased availability of GP work in Ireland, as this decline in the flow of GPs to Australia coincides with a period of increased investment in general practice in Ireland, including the reversal of cuts which had been implemented during the economic recession [58]. The data presented in Table 5 indicates that Australia was a popular destination for Irish citizen GPs especially in 2016–2018.

**Table 5:** Flow of Irish citizen (visa data) and/or Irish-trained (new entrant to medical register) GPs to key destination countries

Country	2012–2013	2013–2014	2014–2015	2015–2016	2016–2017	2017–2018	2018–2019	2019–2020	2020–2021	2021–2022	2022–2023
Australia (visa)^b^	74	81	54	57	70	66	27	30	13	24	22
New Zealand (new entrant)^c^	2	0	2	2	4	2	5	2	3	4	-^a^
	**2012**	**2013**	**2014**	**2015**	**2016**	**2017**	**2018**	**2019**	**2020**	**2021**	**2022**
United Kingdom (new entrant)^d^	3	-	8	6	16	9	6	8	5	9	3
Canada (visa)^e^	12	8	15	14	21	13	8	10	6	5	8
**Total**	**91**	**89**	**79**	**79**	**111**	**90**	**46**	**50**	**27**	**42**	**33**

^a^Data was only requested for a period of ten years from 2012–2021, however in a number of cases the destination countries provided us with data up to the most recent completed year (2023). We have thus presented data up to 2023 in those cases

^b^Department of Home Affairs [37]

^c^New Zealand Medical Council

^d^General Medical Council

^e^Immigration, Refugees & Citizenship Canada

#### New Zealand

In terms of the number of Irish-trained GPs newly registered with the New Zealand Medical Council, in Table 5 there appears to be a discrepancy between the numbers of Irish-trained doctors newly joining the GPregister, and the overall number of Irish-trained doctors registered as GPs. The increases in the overall stock of GPs are slightly larger than the number of new entrants, e.g. flow data shows 3 new entrants to the medical register in 2020–2021, while the stock data show an increase of 7 Irish-trained doctors on the l register 2020–21. Perhaps this indicates that Irish doctors are emigrating to New Zealand as doctors and obtaining GP training once there?

#### United Kingdom

The number of Irish-trained GPs newly entering the General Medical Council register (as GPs) each year 2012–22 is very low (see Table 5).

#### Canada

The number of working visas issued to Irish citizen GPs entering Canada 2012–2022 is also very low (see Table 5).

### Numbers of Irish-trained doctors enrolled in GP training abroad

To explore the apparent discrepancies between stock and flow data in relation to New Zealand and the United Kingdom, we requested data on the number of Irish-trained doctors enrolled in GP training programmes abroad, to ascertain whether Irish or Irish-trained doctors are becoming GPs post- emigration. These doctors may not be recorded as “new entrants” to the GP register as their registration may simply involve a transfer onto the GP register, so they will be recorded in the stock but not the flow data. This could indicate that doctor emigration from Ireland is happening pre-rather than post GP qualification. The number of Irish-trained doctors enrolled in GP training in three countries where data was available, are shown in Table 6 and are relatively high, particularly in the UK.

**Table 6:** Irish-trained doctors enrolled in GP training programmes in the UK, NZ, Australia

	2012	2013	2014	2015	2016	2017	2018	2019	2020	2021	2022	2023
United Kingdom	52	49	49	54	64	62	63	76	96	115	134	-
New Zealand	-	12	9	10	12	15	18	24	24	27	25	21
Australia	-	-	-	-	-	-	-	-	-	-	-	38

## Discussion

Drawing on secondary data from key destination countries, this paper aimed to ascertain the extent to which GP emigration may be contributing to Ireland’s GP crisis. The results are mixed; while there is a large cohort of Irish-trained GPs abroad, there is also a relatively small flow of Irish GPs emigrating from Ireland each year. These results will be discussed in further detail below. We will also reflect on the national and international implications of patterns of GP emigration.

### What does the data tell us about GP emigration and its role in the GP workforce crisis?

The secondary data from key destination countries presented in this paper paints a mixed picture regarding the significance of GP emigration in Ireland’s GP crisis Recent flow data indicates a relatively small outward flow of GPs from Ireland to the key destination countries of the UK, Australia, New Zealand and Canada.

However, if these emigration figures are combined and considered alongside the overall number of GPs trained in Ireland annually, they appear more noteworthy. For example, between 2021 and 2022, 42 Irish trained/Irish citizen GPs emigrated to Australia, New Zealand, Canada or the UK. In the same year, 144 GPs completed training in Ireland. This level of GP emigration relative to the level of GP training is significant as it indicates that 30% fewer GPs than expected entered the Irish healthcare system in that year; that is, the net increase to the stock of GPs in Ireland based on the number of new trainees was 30% lower than expected as it was offset by the emigration of 42 GPs in the same period.

In 2022–23; 33 GPs emigrated to Australia, Canada or the UK (data from New Zealand not available), while 182 GPs completed training in Ireland, representing 18% fewer GPs in the Irish system than expected for that year. These data indicate that GP emigration may have an impact on GP workforce planning in the Irish context and that trends should be monitored and factored into GP workforce planning models going forward. A timely reminder of the importance of keeping abreast of emigration trends is provided by recent policy changes in Australia. In October 2024, Australia introduced a fast-track registration process for Irish-trained GPs [49]. Such policies, introduced to address GP shortages in Australia will potentially have a significant impact on GP emigration patterns between Ireland and Australia and must be monitored and factored into Irish GP workforce planning.

The other international implication of GP emigration from Ireland relates to the extent to which Ireland relies on internationally-trained GPs to staff it’s health system. Figures from 2022 indicate that just under a quarter of GPs working in general practice in Ireland were internationally-trained [45].

Additionally, an examination of the ‘stock’ data reveal a substantial cohort of Irish-trained doctors working in general practice in three destination countries– Australia, New Zealand and the UK. This suggests a historical pattern of GP emigration, particularly to the UK., It may highlight the presence of a cohort of emigrant GPs who could be encouraged to return to practice in Ireland in the future. Policies to encourage return migration of Irish trained GPs could form part of Ireland’s strategy for addressing the GP workforce crisis.

Initial data presented in this paper indicate that some Irish-trained emigrant doctors are opting to complete GP training abroad. For example, in 2022, there were 159 Irish-trained doctors enrolled in GP training programmes in the UK and New Zealand. Further research is required to ascertain whether these doctors complete GP training with the aim of remaining abroad; or if their intent is to return to Ireland as a qualified GP (and whether it is possible to return to practice in Ireland with GP qualifications from these countries).

In general, the data suggests that GP emigration (either before or after completing GP training) is not an insignificant issue in the Irish context, but is one that must be closely monitored in light of increased competition for GPs from countries such as Australia [49]. It is likely that factors other than emigration also play a role in the GP workforce crisis in Ireland. International research on the wider global GP crisis suggests that a myriad of complex factors drive GP shortages including; an ageing workforce insufficient GP training, retention challenges, and increased mobility of healthcare workers [11, 13, 14]. Decreased attractiveness of general practice as a career due to working conditions and lack of satisfaction with work-life balance [9] may also contribute to increased attrition. Indeed, the most recent report from the Medical Council found that 32% of clinically active GPs in Ireland reported working 30 h per week or less [45], suggesting that less than full-time working should also be factored into workforce planning for general practice. The size of the GP workforce required will also depend on the number of GPs opting for less than full-time working and the extent to which it is factored into GP workforce planning.

### The need for better data on GP workforce, GP emigration and GP return

This paper highlights the need for improved routinely available data on the GP workforce. This is important for workforce research, but essential for workforce planning now and into the future. In the context of a GP workforce crisis, Ireland must begin to capture and publish this data to enable the development of an accurate and up to date picture of patterns of GP emigration, GP return and GP retention and to strengthen GP workforce planning. but there is a clear need for a national GP workforce dataset; recording the number of GPs in clinical practice, their age, their working hours and number of sessions worked as well as demographic factors (gender, location etc.) etc. The challenges associated with quantifying the GP workforce in Ireland have been previously noted. A 2015 HSE NDTP report [47] highlighted the paucity of reliable data on the GP workforce in Ireland, particularly in relation to locum and out-of-hours services and recommended the introduction of a national GP register [47]. However, almost a decade later, this recommendation has not been implemented.

There is also a need to track GP emigration, GP retention and GP return on an ongoing basis (the HSE NDTP Annual Medical Retention reports will help to achieve this) so that patterns can be spotted early and policy responses developed in response. If emigration is not currently a major driver of the GP workforce crisis in Ireland, then consistent tracking and collation of data on GP emigration and retention can monitor this and direct attention to those factors which may be playing a larger role, such as less than full time working, early retirement, or attrition from general practice to work in other areas of medicine. To to future-proof the GP workforce and ensure continued access to healthcare Ireland must be able to identify the drivers of this crisis and respond to them in a timely manner.

### Implications for policy

The GP workforce crisis poses a significant risk to the Irish population in terms of their access to healthcare. The crisis also threatens Ireland’s aim to transform into a primary-care centred system of universal healthcare, the proposed ‘Sláintecare’ healthcare reforms [30], which hinges on a strong GP workforce [59]. It has been recognised that the GP workforce needs to be expanded [27]. Although GP training places have been increased and the the GP retirement age extended,, to strengthen the GP workforce, and adapt to the increased demands on primary care, Ireland must also improve GP retention. This requires a better understanding of the underlying causes of the GP workforce crisis in Ireland, and how the crisis is playing out, via emigration, early retirement, attrition and less than full time working. The data presented in this paper illustrates only part of the picture of GP emigration from Ireland, and highlights the need for better data collection at the point of exit, rather than relying on data from the destination countries.

### Limitations

The scope of the paper was limited by the availability of data from destination countries and the paucity of data on emigration available from Irish sources. The researchers also placed data requests to the United States, the United Arab Emirates, Saudi Arabia and Qatar, but were unable to source that data.

A second limitation is that the data collated for this paper is messy; different formats of data from different countries means that the cohorts being compared across countries are nott necessarily the same (Irish-trained versus Irish citizen doctors; see Fig. 1 above). It is worth noting when adding flow data across countries from different sources (medical registration versus immigration), that they are not capturing exactly the same cohorts of doctors, and so any totals are imperfect estimates due to the nature of the data.

## Conclusion

This paper sought to ascertain whether or not GP emigration contributes to Ireland’s GP workforce crisis. The answer is mixed. The data appear to suggest that the outward flow of GPs from Ireland is not large. However, the data also point to the presence of a significant stock of Irish trained/Irish citizen GPs in the UK and Australia. This suggests historical GP emigration from Ireland and highlights the need for better data on the GP workforce and attrition (whether due to emigration, retirement, early retirement or even less than full time working). The approach and methods that we have adopted in this paper may be a useful template for other source countries. Accurate and up-to-date data is an essential tool to ensuring a sustainable GP workforce in to the future. In order to safeguard continued access to primary care in Ireland, and to facilitate the implementation of healthcare policy which seeks to expand the role of primary care, the GP workforce must be monitored in order to identify issues early and intervene before reaching crisis-point.

## Data Availability

All data generated or analysed during this study are included in this published article [and its supplementary information files].

## Abbreviations

WHO: World health organisation
GP: General practitioner
ICGP: Irish college of general practitioners
PMQ: Primary medical qualification
HSE: Health service executive
NDTP: National doctors training and planning
MCI: Irish medical council

## Footnotes

1 In this manuscript, all of the data used in the study is included in the manuscript. The study used aggregate data on the emigration of general practitioners from Ireland, collated from key destination countries; that is, the overall number of GPs from Ireland in each of those countries. These figures are included in the manuscript were obtained on request from the relevant and cited sources and/or found in publicly available datasets such as the Australian Temporary Work Visa Programme dataset, which is fully referenced in the manuscript The tables included in the manuscript are the dataset for this study, and we only present descriptive analyses of these overall numbers. We did not conduct any analyses on raw data, simply compiled overall (total) numbers from a number of key destination countries.
